# Defending the leaf surface: intra- and inter-specific differences in silicon deposition in grasses in response to damage and silicon supply

**DOI:** 10.3389/fpls.2015.00035

**Published:** 2015-02-11

**Authors:** Sue E. Hartley, Rob N. Fitt, Emma L. McLarnon, Ruth N. Wade

**Affiliations:** Department of Biology, University of YorkYork, UK

**Keywords:** plant defense, silicon, abrasion, grasses, leaf surface, phytoliths, spines

## Abstract

Understanding interactions between grasses and their herbivores is central to the conservation of species-rich grasslands and the protection of our most important crops against pests. Grasses employ a range of defenses against their natural enemies; silicon-based defenses have been shown to be one of the most effective. Silicon (Si) is laid down on the leaf surface as spines and other sharp bodies, known as phytoliths, making grasses abrasive and their foliage indigestible to herbivores. Previous studies on Si defenses found that closely related species may have similar levels of Si in the leaves but differ markedly in abrasiveness. Here we show how the number, shape and distribution of Si-rich phytoliths and spines differ within and between different grass species and demonstrate that species also differ in their ability to change the deposition and distribution of these defenses in response to damage or increases in Si supply. Specifically, we tested the response of two genotypes of *Festuca arundinacea* known to differ in their surface texture and three different grass species (*F. ovina, F. rubra*, and *Deschampsia cespitosa*) differing in their abrasiveness to combined manipulation of leaf damage and Si supply. *F. arundinacea* plants with a harsh leaf surface had higher Si content and more spines on their leaf surface than soft varieties. *F. ovina* and *D. cespitosa* plants increased their leaf Si concentration and produced an increase in the number of leaf spines and phytoliths on the leaf surface in response to Si addition. *F rubra* also increased leaf Si content in response to treatments, particularly in damaged leaves, but did not deposit this in the form of spines or increased densities of phytoliths. We discuss how the form in which grasses deposit Si may affect their anti-herbivore characteristics and consider the ecological and agricultural implications of the differences in allocation to Si-based defenses between grass species.

## INTRODUCTION

Grasslands including managed rangelands and pastures cover ~40% of the earth’s surface and grasses are an important plant family agriculturally, economically and ecologically (Strömberg, 2005; Gibson, 2009). Not only are our most widely grown and consumed food crops domesticated grass species, but grasses also provide grazing for both wild and domesticated animals. In their long co-evolution with grazers (Coughenour et al., 1985), grasses have developed a number of defensive strategies to both tolerate and repel herbivory (Vicari and Bazely, 1993), including rapid regrowth ability from their basal meristems (also an adaptation to fire and trampling common in these ecosystems) and a combination of both chemical defenses (including those provided by endophyte mutualists; Hartley and Gange, 2009) and physical defenses (McNaughton and Tarrants, 1983).

One such physical defense is the accumulation of silicon (Si) which has been previously reported to accumulate in high levels in the leaves of many grass species (Hodson et al., 2005), although the amount of Si accumulated shows large inter and intra species variation (Massey et al., 2007a; Soininen et al., 2013). There is clear evidence to demonstrate that these high levels of Si are effective anti-herbivore defenses, with impacts on the feeding preferences and performance of both vertebrate (McNaughton and Tarrants, 1983; Gali-Muhtasib et al., 1992; Massey and Hartley, 2006; Teaford et al., 2006; Massey et al., 2009) and invertebrate herbivores (Goussain et al., 2005; Massey et al., 2006; Kvedaras et al., 2007; Massey and Hartley, 2009).

These adverse effects appear to be mediated at least in part by abrasion: Si is primarily deposited as amorphous silica in the form of solid bodies known phytoliths in the epidermis (Richmond and Sussman, 2003; Currie and Perry, 2007). Phytoliths are hard and often irregular shapes and Si is also deposited in leaf hairs, trichomes and spines; all these structures could influence the texture and abrasiveness of the leaf. It has been suggested that Si abrades the teeth of mammalian herbivores (Jernvall and Fortelius, 2002; Erickson, 2013 but see Sanson et al., 2007) and an increase in leaf abrasiveness has been shown to reduce the performance of both vertebrate and invertebrate herbivores. For example, the amount of mandible wear feeding imposed on African armyworm (*Spodoptera exempta* Walker), and hence the reduction in their ability to extract nitrogen from their food, is correlated with the Si levels of the foliage they consume (Massey and Hartley, 2009), whilst voles prefer, and perform better on, grasses which are less abrasive (Massey and Hartley, 2006; Massey et al., 2008).

Previous work has suggested that foliar Si levels and the abrasiveness of grass leaves are reasonably well correlated: over 70% of the variation in abrasiveness across 18 different grass species was explained by Si content (Massey et al., 2007a). However, Si levels and abrasion are not always closely linked. For example, despite containing similar concentrations of Si, *Festuca ovina* L. was found to have much higher levels of abrasiveness compared to *F. rubra* L., whilst increasing leaf Si concentration through Si addition produced a smaller increase in abrasiveness in *Poa annua* L., a relatively palatable species, than in the more unpalatable *Brachypodium pinnatum* (L.) P. Beauv. (Massey et al., 2007a). It is possible that different grass species deposit their available Si differently at their leaf surfaces, influencing the abrasiveness of their leaves. It is certainly well-known that phytolith morphology varies between plant taxa, with differences between species sufficiently marked and consistent to allow phytoliths to be useful in palaeobotany (Strömberg, 2005). Some phytoliths are relatively smooth in shape, others much less so and it seems likely that the size, shape and density of phytoliths and Si rich spines will influence the abrasiveness of the leaf surface and its impact on the preferences and performance of herbivores.

Another influence on the nature and effectiveness of the leaf surface defenses will be the amount of Si available in the soil to take up and deposit (Currie and Perry, 2007). Previous exposure to herbivory has also been shown to impact on the levels of Si-based defenses in plants. It has long been known that Si levels increase in grasses from grazed areas (McNaughton and Tarrants, 1983) and herbivore-specific induction in Si defenses has been shown to occur, but only after repeated damage above a threshold (Massey et al., 2007b; Reynolds et al., 2012). More recently it has been shown that there are differences in both grass species and grass genotypes in the extent to which they respond to damage with increased Si uptake (Soininen et al., 2013).

The aim of this study was to determine the leaf Si concentration of different forage grass genotypes and naturally occurring grass species previously reported to differ in their leaf abrasiveness (see below), and to investigate whether these differences in leaf texture are related to the way Si is deposited on the leaf surface, potentially influencing the effectiveness of their use of Si in terms of reducing palatability to herbivores. We hypothesized that:

(i) harsher and more abrasive species and varieties would have higher leaf Si levels than softer ones;
(ii) species with similar Si levels which differed in abrasiveness would do so because they used their Si to produce a greater number of phytoliths and/or spines on their leaf surface, and that these spines would be larger or sharper.

We also hypothesized that irrespective of grass species, foliar Si levels would be elevated by increases in Si supply, and hence in potential uptake (Epstein, 1999; Cooke and Leishman, 2011), and by damage, due to induction (Massey et al., 2007b). We also expected foliar Si levels to be highest in plants receiving both Si addition and damage, since induction in response to damage would be able to capitalize on the additional Si available in the soil. We predicted that the most abrasive species would deposit this additional Si in the form of surface spines to a greater extent than less abrasive species.

## MATERIALS AND METHODS

### STUDY SPECIES

*Festuca arundinacea* Schreb. is a cool season perennial grass (Gibson and Newman, 2001) and a dominant pasture and turf grass in North America, Australia and Europe (Hand et al., 2012). It has a number of attractive agronomic attributes, including high yields, winter persistence (Gibson and Newman, 2001) and tolerance to drought (Cougnon et al., 2014), though it appears to be relatively unpalatable to cattle. In mixed culture fields, cattle rarely choose it as their forage choice (Gibson and Newman, 2001), possibly because of the “harsh” (i.e., feeling rough to the touch) texture of the leaf surfaces. There is interest amongst forage breeders in understanding the basis of this leaf harshness and unpalatability to improve the attractiveness of this species as forage. A number of varieties of *F. arundinacea* ranging from very harsh to very soft leaf textures have been developed by plant breeders (based on manual evaluation of surface roughness in the field by plant breeders), enabling the testing of the hypothesis that Si has a role in causing the harsh leaf surfaces.

We can also address the relationship between Si content and leaf texture by exploiting the natural variation in the relationship between Si and abrasion across native non-forage *Festuca* species: *F. ovina* and *F. rubra* may differ so markedly in their leaf abrasion despite similar foliar Si levels (Massey et al., 2007a) because of the way they utilize the Si they take up. Specifically, *F. ovina* may produce a greater number, larger or more abrasive spines and phytoliths than *F. rubra*. We compared these species with the Si content and leaf texture of *Deschampsia cespitosa*, a grass known to be particularly unpalatable to herbivores due to its Si defenses (Massey and Hartley, 2006, 2009).

### PLANT GROWTH AND EXPERIMENTAL TREATMENTS

The two varieties of *Fesctuca arundinacea* were grown individually from seed in a loam based compost (John Innes No.2) in 13 cm pots. Both varieties were harvested 8 weeks after sowing at the point where all plants had at least four tillers. *F. ovina, F. rubra*, and *D. cespitosa* (L.) were grown from seed individually in peat based F2 (Levington, Scotts) compost in 10 cm diameter pots in the greenhouse conditions 16 h daylight, 20°C/15°C day/night. Due to their slower growth rates in relation to *F. arundinacea*, these three species were harvested 18 weeks after sowing.

Once established, plants were randomly assigned to four treatments: control plants with no Si addition and no damage (Si- D-), Si addition only (Si+ D-), damage only (Si- D+), and both Si addition and damage (Si+ D+). Treatments were imposed 3 weeks after sowing in the case of *F. arundinacea*, with plants harvested 5 weeks later, and 8 weeks after sowing in the case of the other three species, which were harvested 10 weeks later. There were six replicate plants of each treatment combination for *F. arundinacea* and seven replicate plants of each treatment for the other species.

For all grass species and varieties, Si addition was achieved by watering plants with 150 mg L^-1^ solution of dissolved sodium metasilicate (Na_2_SiO_3_⋅9H_2_O). Plants were watered 100 ml twice a week with either Si solution or deionised water. *F. ovina, F. rubra*, and *D. cespitosa* plants in the two treatments where damage was applied were mechanically damaged using scissors once a week over 10 weeks. Half of the plant’s leaves were damaged by removing approximately half the leaf lamina down the midrib; the remaining leaves were left undamaged. When damaged plants were harvested, damaged and undamaged leaves were kept separated for Si analysis in order to test for induction of Si defenses in both the undamaged and the damaged leaves on the damaged plants.

### Si ANALYSIS BY PORTABLE X-RAY FLUORESCENCE SPECTROMETRY (P-XRF)

Silicon was analyzed by P-XRF, calibrated using Si-spiked synthetic methyl cellulose and validated using Certified Reference Materials of NCS ZC73014 ‘Tea’ obtained from China National Analysis Center for Iron and Steel (Reidinger et al., 2012).

Both P-XRF and EDX (see below) work on the principle of excitation of inner orbital electrons by an X-ray radiation source. As the excited electrons relax to the ground state, they fluoresce, thereby ejecting photons of energy and wavelength characteristic of the elements present and their concentrations. XRF instruments are widely used for the non-destructive, rapid and accurate elemental analysis of a range of materials (Jang, 2010).

Leaf material was ball milled (Retsch MM 400, Haan, Germany) for 2 min at a vibrational frequency of 24 Hz (60 min^-1^) with two 1 cm diameter steel grinding balls in a 25 ml grinding jar. Leaf material was pressed at 11 tons for approximately 5 s into 5 mm thick cylindrical pellets with a manual hydraulic press using a 13 mm die (Specac, Orpington, UK). Si analysis (% Si DW) was performed using a commercial P-XRF instrument (Nitron XL3t900 GOLDD analyser: Thermo Scientific Winchester, UK) held in a test stand (SmartStand, Thermo Scientific, Winchester, UK).

### SURFACE ANALYSIS BY SCANNING ELECTRON MICROSCOPY (SEM) AND ENERGY DISPERSIVE X-RAY SPECTROSCOPY (EDX)

Leaf samples were taken from two replicate plants per species from all four treatment combinations for the inter-species experiment. A square section (~5 mm^2^) of leaf material either side of the midrib of a mature, expanded leaf blade on the main stem was cut with a razor blade and immediately placed in fixative (2.5% glutaraldehyde, 4% formaldehyde in 100 mM phosphate buffer). For the *F. arundinacea* experiment, samples were taken from the harsh variety with added Si and the soft variety with no added Si (Si addition had no effect on Si levels in this experiment – see below). A square section (~1 cm^2^) spanning the entire width of the mature, expanded leaf blade was cut from the main stem for each variety. The samples were then dehydrated through an acetone graduated series (samples were placed at 25, 50, 75, and 100% acetone concentration for ~1 h) and critical-point dried. Samples were then mounted on sticky carbon tabs and coated with 8nm thick layer of platinum-palladium.

SEM images were obtained using FEI Sirion S-FEG FESEM (Oxford Instruments, Tubney Woods, Abingdon, Oxfordshire). EDX was used in conjunction with the SEM to determine the elemental composition of the samples; an electron beam was focused on the samples and the difference between the ground state (unexcited state) and the excited state was measured by the energy-dispersive spectrometer which determines the elements present in the sample (Goldstein, 2003). The EDX analysis was performed using an Oxford INCA analysis system FESEM (Oxford Instruments, Tubney Woods, Abingdon, Oxfordshire), using the working distance of 10 mm. For the SEM images, the voltage was 5–10 kV and for the EDX analysis the voltage was 12 kV.

### STATISTICAL ANALYSES

All analyses were performed using R (version 3.0.2). ANOVA was used to test the main and interactive effects of grass species or genotype, Si addition and damage treatments on leaf Si concentrations. The effects of the Si and damage treatments were assessed on undamaged leaves from plants across all four treatments, to test if damage led to increased Si levels systemically in damaged plants in comparison with undamaged plants. A separate analysis tested for the effect of these treatments in damaged leaves from damaged plants compared to undamaged leaves from undamaged plants. *Post hoc* Tukey contrast tests were performed using the ghlt function from multcomp package (Hothorn et al., 2014).

Linear models were used to check for normality and homogeneity of variance following Crawley (2007). Si (%) values were transformed using the arcsine squareroot transformation to meet the assumptions of the test. Significance was set at *P* < 0.05 for all analyses.

## RESULTS

### INTRASPECIFIC DIFFERENCES

**FIGURE 1:**
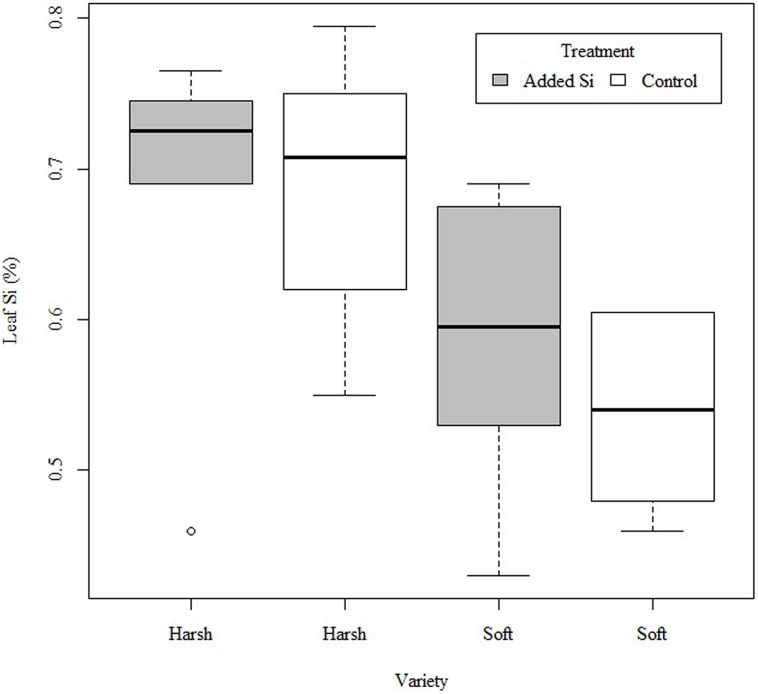
**Silicon (Si) concentration (%) of harsh and soft variety of *Festuca arundinacea* with no Si addition (control) or Si addition (+Si)**.

The *F. arundinacea* variety with a harsh leaf surface texture had significantly higher leaf Si concentration than the soft texture variety (*F*_1,19_ = 8.586, *P* < 0.01), but there was no significant interaction between Si addition and variety (*F*_1,19_ = 0.282, *P* > 0.5; **Figure 1**).

**FIGURE 2:**
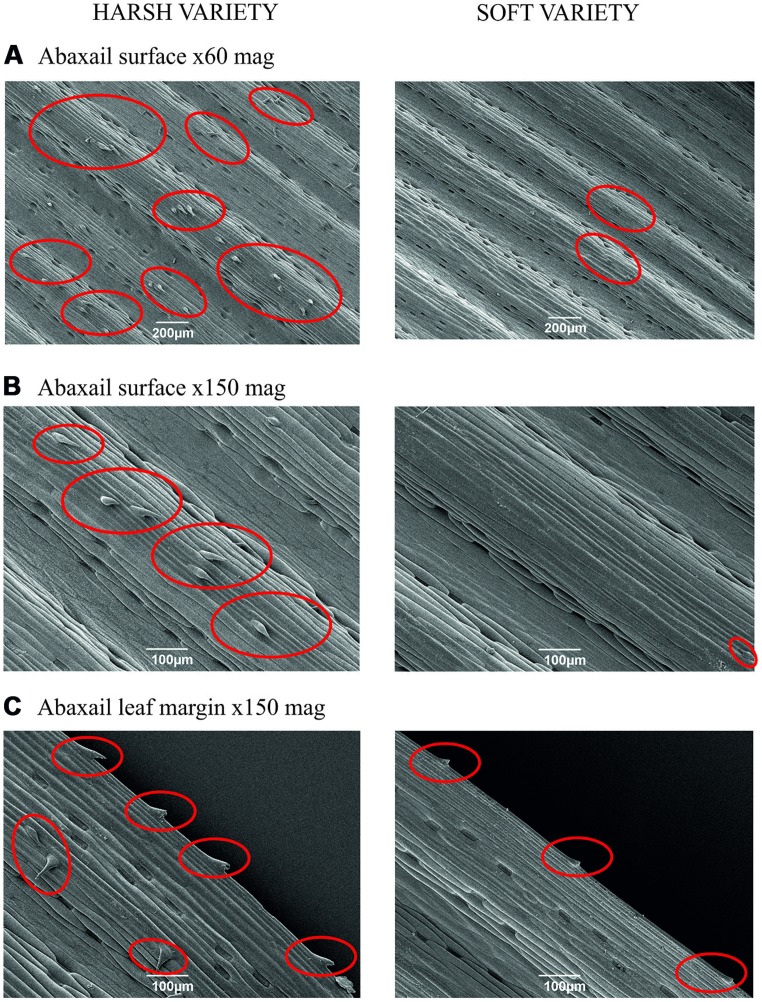
**(A)** Harsh and soft variety abaxail surface (x60 magnification), **(B)** Harsh and soft variety abaxail surface (x150 magnification), **(C)** Harsh and soft variety abaxail margin (x150 magnification). Red circles indicate leaf spine presence.

The two main types of cells which were silicified were leaf spines (trichomes) and silica short cells. The harsh variety had more spines present on the abaxial surface than the soft variety (**Figures 2A,B**), which not only had fewer spines but the spines which were present were smaller and had a different morphology (**Figures 2B,C**). The spines present on the harsh variety were bigger in size and the point of the spines were spear-like in appearance; these spines also appeared to protrude more from the surface compared with the soft variety, where the spines were smaller in size and the points of the spines lay closer to the surface of the leaf. No spines were observed on the adaxial surface of either variety of *F. arundinacea* (images not shown).

**FIGURE 3:**
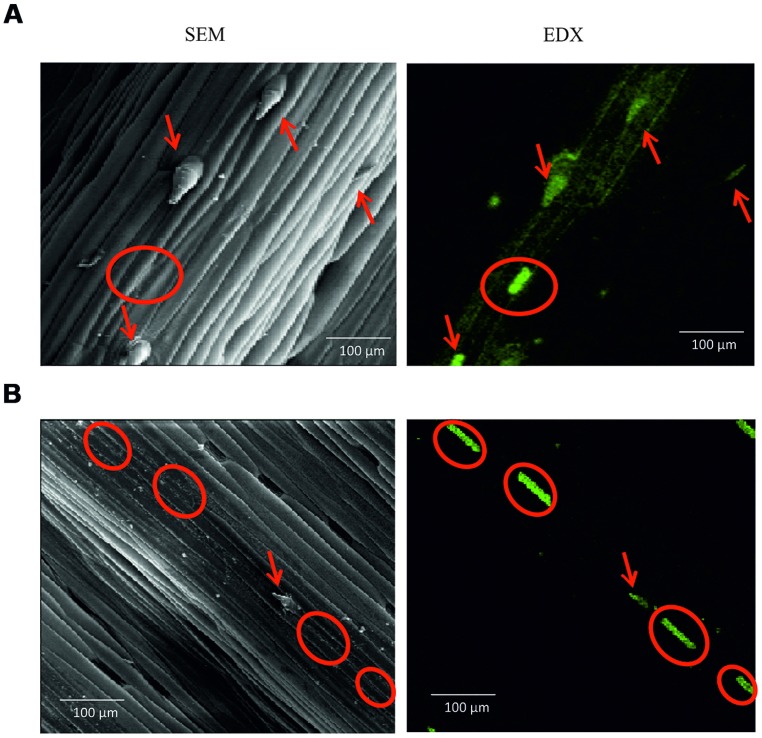
**(A)** Harsh variety abaxail surface (x200 magnification), **(B)** Soft variety abaxail surface (x200 magnification). Scanning electron microscope (SEM) represented by grey images. Electron density X-ray spectroscopy (EDX) represented by black and green images; green intensity indicates Si concentration. Red circles indicate trichomes with Si deposition. Red arrows indicate silica short cells.

The spines were rich in Si (**Figure 3**), and there were other Si deposits on the leaf surface in the form of silica short cells. Generally, the harsh variety had a greater over surface deposition of Si compared with the soft one, depositing the Si within the leaf spines (red circles **Figure 3**), and also silica short cells surrounding the spines (red circles **Figure 3**). The soft variety deposited Si as silica short cells on the leaf surface within fewer, smaller leaf spines containing less Si than in the harsh variety (**Figure 3B**).

### INTERSPECIFIC DIFFERENCES

**FIGURE 4:**
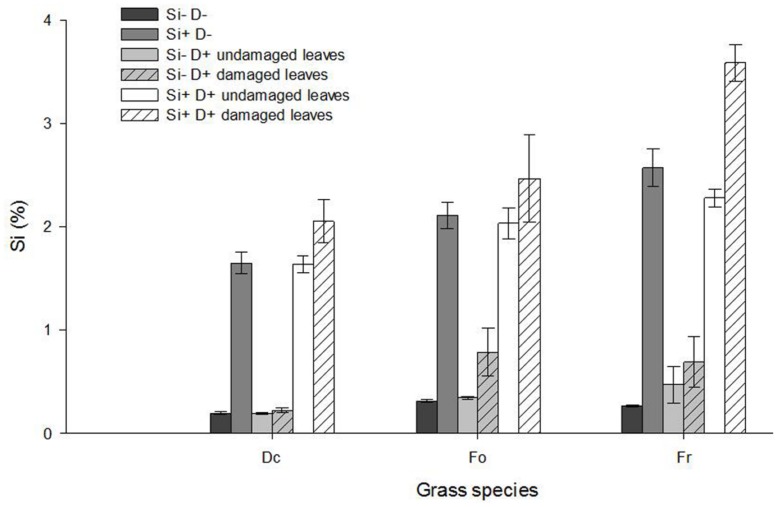
**Leaf Si concentration (%) of *D. cespitosa* (Dc), *F. ovina* (Fo) and *F. rubra* (Fr) plants treated with no Si addition and no damage (Si- D-), Si addition only (Si+ D-), damage only (Si- D+), and both Si addition and damage (Si+ D+).** Values represent mean ± SE bars of seven replicates.

Grass species differed in their leaf Si concentration (**Figure 4**; Spp effect: *F*_2,72_ = 23.62, *P* < 0.001 undamaged leaves; *F*_2,72_ = 15.99, *P* < 0.001 damaged leaves) with significantly lower Si concentrations in *D. cespitosa* compared to *F. rubra (post hoc* Tukey tests *P* < 0.05 for both undamaged and damaged leaves) and *F. ovina (post hoc* Tukey tests *P* < 0.01 for both undamaged and damaged leaves*).* Plants treated with Si addition responded with an increase in their leaf Si concentration irrespective of whether leaves were damaged or not (Si effect *F*_1,72_ = 1265.33, *P* < 0.001 undamaged leaves; *F*_1,72_ = 463.17, *P* < 0.001 damaged leaves). In comparison with undamaged leaves on undamaged plants, damage did not increase Si levels in undamaged leaves on damaged plants (*F*_1,72_ = 0.03, *P* > 0.05 NS), but there was a significant increase in the Si levels in the damaged leaves (*F*_1,72_ = 17.92, *P* < 0.001), suggesting that damage-induced increases in Si levels are localized in damaged leaves and do not spread to undamaged ones on the same plant (**Figure 4**).

In the case of undamaged leaves there was no significant interaction between the effects of species and Si addition on Si levels (*F*_2,72_ = 2.89, *P* > 0.05 NS), suggesting all three species responded in a similar way to increases in Si supply in terms of the allocation of this additional Si to their undamaged leaves. However this was not the case for damaged leaves, where a significant Species × Si addition interaction (*F*_1,72_ = 4.62, *P* < 0.05) suggests species differ in where they allocate any additional Si once they are damaged. This is confirmed by the *post hoc* Tukey tests which showed that *F. rubra* had significantly higher concentrations of Si in damaged leaves under conditions of increased Si supply than either *F. ovina* (*P* < 0.01) or *D. cespitosa* (*P* < 0.001; **Figure 4**).

**FIGURE 5:**
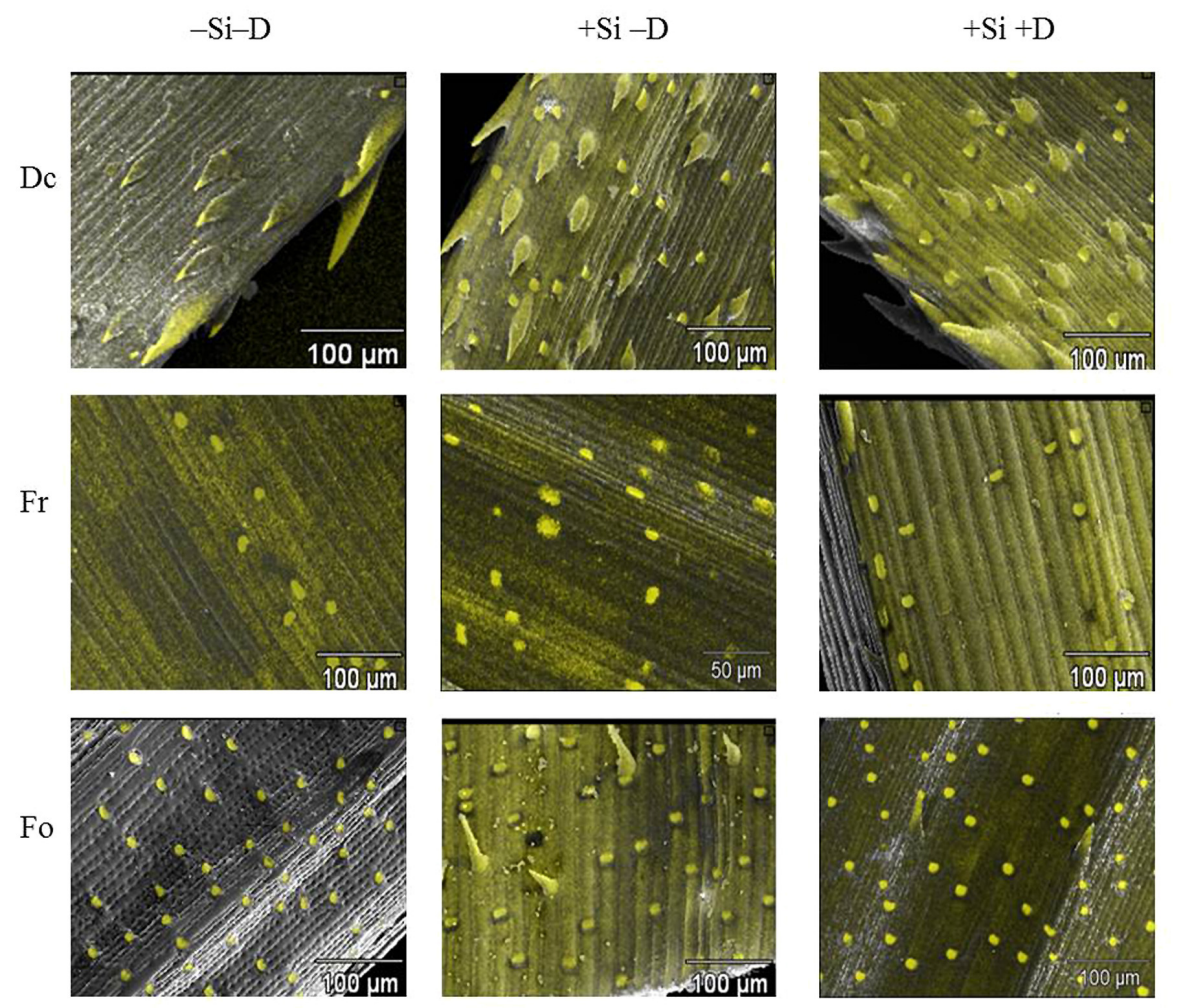
**Images (x300 magnification) of *D. cespitosa* (Dc), *F. ovina* (Fo), and *F. rubra* (Fr) plants treated with control (Si- D-), Si addition with no damage (Si+ D-) and Si addition and damage (Si+ D+).** Yellow intensity indicates Si concentration. Red circles indicate trichomes with Si deposition. Red arrows indicate silica short cells.

The SEM revealed differences in Si deposition on the leaf surfaces of the grass species (**Figure 5**). The leaf surface of *D. cespitosa* plants was found to have abundant Si-rich leaf spines (trichomes), even in the absence of Si addition. In contrast, the leaf surface of *F. rubra* and *F. ovina* plants growing without added Si had only rounded silica short cells and no leaf spines, although the round phytoliths were much more prominent and frequently distributed on the leaf surface of *F. ovina* than *F. rubra*. Both *F. ovina* and *D. cespitosa* plants deposited additional phytoliths (silica short cells) in response to increased Si supply, especially in the presence of damage, but Si addition had very little effect on the number or shape of the phytoliths deposited on the leaf surface of *F. rubra*. The damage alone treatments had little effect on leaf surface Si deposition in any of the grass species (images not shown).

## DISCUSSION

Grass species and varieties differed in their leaf Si concentrations and the form in which this Si is deposited on the leaf surface. The more unpalatable and abrasive species, namely *D. cespitosa* and the harsh variety of *F. arundinacea* had both larger and a greater abundance of Si-rich spines compared to the more palatable *F. rubra* and soft variety of *F. arundinacea*. Si addition resulted in an increase in leaf Si concentration in three out of the four grass species and altered the deposition of Si on the leaf surface in the case of *D. cespitosa* and *F. ovina*, but had little impact on the surface of *F. rubra*. The different forms in which Si is deposited at the leaf surface in *F. ovina* and *F. rubra* may explain previous observations that they differ in abrasiveness even though, as we found here, they contain similar amounts of Si. Generally, damage caused a small increase in leaf Si concentration, but did not have a large effect on the form in which the Si was deposited on the leaf surface.

### INTRASPECIFIC DIFFERENCES

As we hypothesized, the harsh variety of *F. arundinacea* had a higher leaf Si level than the soft variety, though our prediction that Si addition would increase foliar Si content was not supported in this species. The differences in foliar Si content between the harsh and soft varieties suggest that Si is contributing significantly toward the differences in leaf texture between them. The harsh variety had significantly higher leaf Si content than the soft variety as well as having a different leaf surface morphology, suggesting that increased levels of Si support the production of increased number and size of leaf spines. This is significant in the context of forage grass: differences in the patterns of deposition of Si on the leaf surface between varieties may offer scope for plant breeders to select for more palatable forage.

Scanning electron microscopy images also revealed that the harsh variety of *F. arundinacea* had more numerous and larger leaf spines than those on the leaf surface of the soft variety, and this was particularly noticeable at the leaf margin. These spines may act as a deterrent to herbivores, especially cattle as they use their tongues to wrap around the blades of grass prior to chewing; if the grass feels spiny then it is likely to seem unpalatable to the cattle. An impact of Si levels on the bite rate of ruminants has been demonstrated experimentally for sheep (Massey et al., 2009), suggesting Si does impair food processing time. The soft variety had far fewer and smaller spines, suggesting these plants are depositing Si in a different way to the harsh variety. Even when not deposited as abrasive spines, Si can still make plants hard to digest, if for example these deposits prevent herbivores crushing cells to extract nutrients, as hypothesized by Hunt et al. (2008).

Contrary to our predictions, neither variety responded to the Si addition treatment with an increase in leaf Si content. This may be related to the young age of the plants and relatively short duration of the Si addition treatment. Si accumulation is influenced by transpiration rates, where older leaves are found to have significantly more Si than younger leaves, primarily due to Si translocation via the transpiration stream (Piperno, 2006). Furthermore, once deposited Si is not remobilized (Richmond and Sussman, 2003), meaning foliar Si levels increase with both plant and leaf age (Reynolds et al., 2012). Although the mechanisms underpinning Si uptake and distribution in plants are still not fully understood, it has been demonstrated that plant species differ in the Si uptake ability of their roots and in the density of their root transporters, as well as in their capacity to upload Si to the xylem (Ma and Yamaji, 2006). More recently, work on rice has suggested that shoots control the regulation of the Si transporters in the root and hence how much Si is taken up into the shoot (Yamaji and Ma, 2011). A study assessing the uptake abilities of over 500 plant species (Ma and Takahashi, 2002) classified them into high, intermediate and non- accumulators and it may be that *F. arundinacea* physiology is such that it is not a high accumulator of Si, even under conditions of high Si supply. It does however, appear to be able to use the Si it does take up very efficiently in terms of spine production, at least in the case of the harsh variety. The mean Si values reported here for *F. arundinacea* are lower than those reported for this species in Hodson et al. (2005), which may reflect differences in age of the plants when sampled or the growing conditions of the plants. However the variation in foliar Si content shown in this species and indeed in other taxa within the genus (Hodson et al., 2005; Massey and Hartley, 2006; Massey et al., 2006, 2007a, 2009) suggests a high degree of phenotypic plasticity in the levels of Si seen within the leaves of *Festuca* species. This is perhaps unsurprising given the numerous factors, including plant genotype, biotic stresses such as herbivory and abiotic ones such as water availability, known to affect Si levels in plants (Soininen et al., 2013; Quigley and Anderson, 2014).

### INTERSPECIFIC DIFFERENCES

Our hypothesis that abrasive species would have higher foliar Si concentration than less abrasive species was not well-supported: *D. cespitosa* has previously been reported to have high leaf Si and to be more abrasive than either of the *Festuca* species, but in this study had lower leaf Si concentration than *F. ovina* and *F. rubra*. Differences in experimental conditions, and hence in plant growth rate, and in plant age, size, and genotype (Soininen et al., 2013) may explain changes in the relative Si concentrations between species, but it is clear that our second hypothesis, namely that more abrasive species had larger, sharper and/or a greater number of spines and phytoliths is supported (**Figure 5**). SEM images revealed that *D. cespitosa* leaves are covered in Si-rich leaf spines which were absent from the leaf surfaces of *F. rubra* (and from *F. ovina* in the absence of additional Si). This strongly suggests that the leaf spines were significantly influencing the abrasiveness of *D. cespitosa* and that phytolith morphology may be more important than leaf Si concentration in determining the abrasiveness of leaves and thus the effectiveness of anti-herbivore defense.

There was a change in morphology and an increase in the number of Si-rich bodies deposited on the leaf surface of *D. cespitosa* and *F. ovina* when plants were provided additional Si; in the case of *D. cespitosa*, phytoliths which had not been present in control leaves were deposited (silica short cells), whereas for *F. ovina*, new, Si-enriched spines were produced, again when spines were not apparent on control leaves. This suggests that these plant species have the ability to deposit new types of Si-based structures to potentially increase their anti-herbivore defenses, whether via abrasion, digestibility effects or both, when Si supply is increased. These changes were most obvious when leaves were also damaged, although interestingly damage in the absence of additional Si did not produce them. In addition to changes in the nature of the spines, the EDX demonstrates that *D. cespitosa* deposited Si only at the tips of spines under control conditions, but under the Si addition treatment, the spines contain Si throughout and the leaf surface is also heavily silicified. A similar pattern was observed in *F. ovina* (**Figure 5**).

Our results support our predictions about the influence of Si supply on the level of Si-based defenses (also see Garbuzov et al., 2011), but damage had less effect on Si-based defenses than we predicted. Although damaged plants were found to have an increase in leaf Si concentration, this was far smaller than the effect of Si supply and there was little effect of damage on spine formation. This may reflect the fact that we used a clipping treatment; simulated damaged may not bring about the same response in spine/phytolith morphology and Si accumulation as herbivory, as reported in previous studies (Massey et al., 2007b). *F. rubra* demonstrated a greater Si uptake to damaged leaves on damaged plants than other species did, which suggests plant species show differences in the way they distribute their Si between different plant parts in response to damage (and potentially other stresses). The mechanism for this is currently unknown, but there have been reports of between species differences in the ability to load Si into the xylem (Ma and Yamaji, 2006).

## CONCLUSION

There were marked differences in the way that even grass species from the same genus deployed the Si they take up in terms of its deposition in structures likely to affect their anti-herbivore defenses. Differences in the localization and the Si-based structures formed has been demonstrated before between plant families (Currie and Perry, 2007), but to our knowledge this is the first time such striking variation has been observed between grass species from the same genus. *F. rubra* had the highest foliar Si content and deposited more Si in damaged leaves than the other two species when plants were damaged under conditions of increased Si supply. However, it is the least abrasive species, presumably because its Si is deposited smoothly and evenly on the leaf surface and not in spines, and any phytoliths produced are few in number and, in contrast to spines, do not protrude substantially above the leaf surface. *D. cespitosa* has a very different strategy: a lower foliar Si content which was less affected by damage and Si addition, but what Si was present was deposited in numerous large spines, particularly at the tip, and under conditions of high Si supply, in a high density of additional structures which are absent under low Si supply. These structures may explain why this species has frequently been shown to be abrasive and unpalatable. Our results suggest that quantifying leaf Si concentration will not give a complete understanding of Si-based anti-herbivore defenses; rather examining how that foliar Si is deposited on the leaf surface will provide a better knowledge of how different plants use their Si and its likely impact on herbivores.

## Conflict of Interest Statement

The authors declare that the research was conducted in the absence of any commercial or financial relationships that could be construed as a potential conflict of interest.
